# Core versus diet-associated and postprandial bacterial communities of the rainbow trout (*Oncorhynchus mykiss*) midgut and faeces

**DOI:** 10.1242/bio.034397

**Published:** 2018-05-18

**Authors:** Eleni Mente, Eleni Nikouli, Efthimia Antonopoulou, Samuel A. M. Martin, Konstantinos A. Kormas

**Affiliations:** 1 Department of Ichthyology and Aquatic Environment, School of Agricultural Sciences, University of Thessaly, 384 46 Volos, Greece; 2 School of Biological Sciences, University of Aberdeen, Aberdeen, AB24 2TZ, UK; 3 Laboratory of Animal Physiology, Department of Zoology, School of Biology, Aristotle University of Thessaloniki, 541 24 Thessaloniki, Greece

**Keywords:** *Oncorhynchus mykiss*, Rainbow trout, Gut, Faeces, Bacteria, Diet

## Abstract

This study investigated the impact of different dietary ingredients, with different protein/lipid sources, on midgut and faeces bacteria community structures just before feeding and 3 h after feeding a single meal to individual rainbow trout (*Oncorhynchus mykiss*). Fish were kept in experimental rearing facilities and fed *ad libitum* twice daily for 5 weeks. Fish were fed three different commercial diets, which contained variations of high or low marine fishmeal/fish oil content. DNA was extracted from midgut and faeces samples for analysis of their bacterial 16S rRNA gene diversity by targeting the V3-V4 region with 454 pyrosequencing. A total of 332 unique bacterial operational taxonomic units (OTUs) were revealed in all samples. However, each sample was dominated (>80% relative abundance) by 2–14 OTUs, with the single most dominant OTU having >30% dominance, indicating that only a few bacteria were fundamental in terms of relative abundance in each treatment. Fifteen OTUs occurred in all samples (core microbiota). The majority of these OTUs belonged to the Proteobacteria, Firmicutes or Tenericutes, and were associated with other animal gut environments. The faecal material and the midgut samples had few overlaps in their shared OTUs. A postprandial response in the gut bacterial community structure 3 h after feeding highlights how dietary stimulation induces structural changes in the microbiota profiles in the established gut bacteria. This study showed that feeding *O. mykiss* different diets and even single meals lead to perturbations in the established gut bacteria of *O. mykiss*.

## Abstract (precis)

**Summary:** The gut bacterial microbiome of rainbow trout contains a few core bacterial taxa and has little overlap with its faeces. Bacterial communities can change even 3 h after feeding.

## INTRODUCTION

The gastrointestinal tract microbiota (GIT) in humans is nowadays considered an integral part of their host's nutritional and immunity machinery (Findley et al., 2016; Li et al., 2016; Mu et al., 2016). This concept has been proved essential in triggering similar scientific research for the GIT in animals. Bacteria are the most important group of the GIT microbiome and, in animals, consist of indigenous or resident cells *sensu* Berg (1996). The communities of the GIT microbiome remain stable over long periods of time and have a global occurrence in the gut, also known as the ‘core microbiome’. Other GIT bacteria are characterised as transient or non-indigenous bacteria, originating mostly from the surrounding environment (e.g. Ringø and Olsen, 1999; Zarkasi et al., 2017), including potentially pathogenic microorganisms and microbes with opportunistic/accidental occurrence in the gut. The GIT bacteria can be found either internally (endobionts) or externally (epibionts) in tissue cells or, alternatively, can be associated with the gut material. Epi- and endobionts are most likely to be part of the ‘resident’ microbiota while gut material associated microorganisms are characterised as ‘transient’. The GIT bacteria assemble in distinct communities, controlled mostly by the food ingested (David et al., 2014) and environmental factors. These communities can be disturbed by bottom-up or top-down perturbations, i.e. mostly inorganic nutrients or organic substrates and grazing or viral controlled mechanisms, which are expressed as significant changes in the bacterial communities’ structures (Lozupone et al., 2012; Mu et al., 2016). Thus, the comparison of different GIT bacterial communities undergoing various nutritional changes can provide the framework for setting the functional role of these microorganisms.

The knowledge of fish GIT microbiomes has progressed considerably over the past three decades, as we have passed from the rather restricted culture based approaches (e.g. Cahill, 1990) to the analysis of the structural (Sullam et al., 2012; Ringø et al., 2016) and also functional (Clements et al., 2014) diversity of fish GIT microbiomes. Moreover, the interest in fish GIT microbiomes lies both in natural (e.g. Givens et al., 2015) as well as commercially reared populations (e.g. Kormas et al., 2014). For the latter, the main interest focuses on the effect of different diets on GIT microbiota and the subsequent impacts on fish growth and health (Ringø et al., 2016). In addition, studies in salmonids have demonstrated changes in gut microbiota in terms of bacterial diversity and abundance influenced by different dietary treatments (Nyman et al., 2017; Lyons et al., 2017; Michl et al., 2017; Huyben, 2018a,b).

The rainbow trout (*Oncorhynchus mykiss*) is one of the main commercially important species in aquaculture. Its GIT microbiome in healthy individuals, with direct or indirect association to the fish's nutrition, has been the focus for almost three decades (Cahill, 1990; González et al., 1999). However, the majority of these studies used culture-dependent methods and, thus, they provide limited insights on true GIT microbiome diversity. More recently, few studies have become available after applying culture-independent approaches, which are far more informative regarding GIT microbiome diversity (Spanggaard et al., 2000; Pond et al., 2006; Kim et al., 2007; Navarrete et al., 2012; Geurden et al., 2014; Ingerslev et al., 2014; Lowrey et al., 2015; Lyons et al., 2017). However, the benefits offered by next-generation sequencing (NGS) are now enhancing our knowledge of fish GIT microbial community structure (Ingerslev et al., 2014; Lowrey et al., 2015; Lyons et al., 2017; Dehler et al., 2017a,b; Huyben et al., 2018a,b Michl et al., 2017; Nyman et al., 2017) for humans and other animals. NGS not only allows a thorough approximation of the diversity of bacterial species, but also a deeper insight into community structural changes at temporal/spatial scales after natural/technical perturbations (Widder et al., 2016). Such microbial community structural changes are informative regarding the habitat where they are found, because bacteria are very responsive to environmental changes (Konopka et al., 2015), especially in the gut, which is a fairly stable environment; dietary supply is the prime nutritional factor for these microorganisms (Flint et al., 2007; Coyte et al., 2015).

Post-prandial changes in protein synthesis in aquatic animals determine a large proportion of specific dynamic action and are influenced by nutritional factors including feed intake and diet composition (Houlihan et al., 1993; Carter et al., 2001). However, very little information is available on postprandial bacterial communities and the effect of time after feeding on fish gut bacterial communities.

In this study, we investigated the impact of feeding low and high marine-based diets on the gut and faeces bacterial community structures of rainbow trout just before feeding and after a single meal. The tested diets had marine ingredients of different protein and lipid compositions. We hypothesised that a 3 h postprandial sampling time was needed to follow the peak of the protein synthesis rates after a single meal in trout (Carter et al., 2001). We aimed at (a) revealing the rainbow trout midgut core bacterial communities, i.e. bacteria occurring in the midgut regardless of the supplied diet, (b) investigating the stability or adaption of the midgut bacterial community structure after a single meal with a specific diet and (c) assessing postprandial bacterial community changes as a test of nutritionally similar diets with different ingredient compositions. We observed in-depth bacterial community structures and their inferred ecophysiological role by using 454 pyrosequencing analysis of the 16S rRNA gene diversity in the epibionts of rainbow trout individual midgut samples.

## RESULTS

### Growth performance

At the beginning of the trial, there were no significant differences among tanks for the initial weight of the fish (45.0±1.11 g, *P*>0.05). Fish final weight was 65.7±2.9 g for Diet A, 54±2.9 g for Diet B and 32.60±1.91 g for Diet D. Food consumption was not measured but fish collected for midgut microbiota analysis had eaten since their stomachs and guts were full.

### Community structure of the midgut microbiota

The total number of reads per treatment, i.e. the sum of all individual replicates, ranged between 3,577 and 8,944 sequences (Table 1). A total of 332 unique bacterial operational taxonomic units (OTUs) were found in this study in all samples. The number of OTUs per treatment ranged between 37 for Diet D at 0 h for faeces (D0f) and 155 for Diet A at 0 h for the gut (A0g). However, the number of dominant OTUs, i.e. cumulative relative abundance of >80% per treatment, varied between 2 (A0f and D3g) and 14 (D3f). In all treatments, the single most dominant OTU (Table 1; Table S1) had relative abundance of 30.0–67.0%.

**Table 1.**
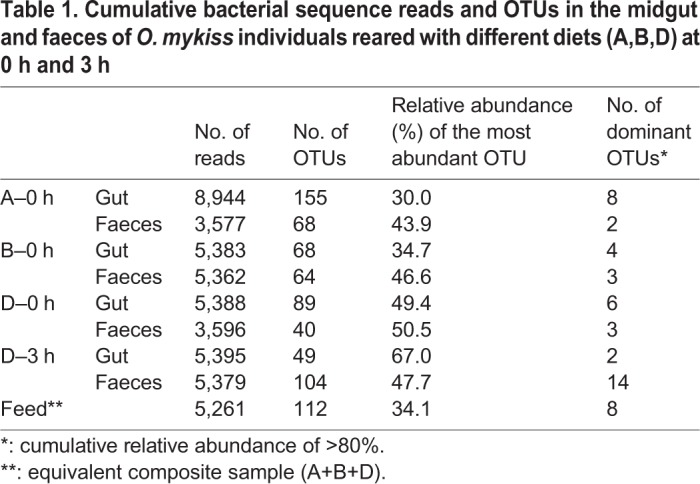
**Cumulative bacterial sequence reads and OTUs in the midgut and faeces of *O.mykiss* individuals reared with different diets (A,B,D) at 0 h and 3 h**

The issue of individual variability has already been recognised in human gut microbiome studies (Bashan et al., 2016) and in fish GIT bacteria (Holben et al., 2002; Hovda et al., 2007; Zarkasi et al., 2017). Indeed, in this study, the coefficient of variation of the OTUs’ average abundance in the triplicated samples reached up to 158.5%, with most OTUs having values >80% (data not shown). Since there were no statistical differences (*t*-test *P*>0.01) between the slopes of the average and total reads’ Rank Abundance Curves, we used the total reads (pooled) of the replicated samples per treatment instead of the average for all further analysis (Besemer et al., 2012).

**Fig. 1.**
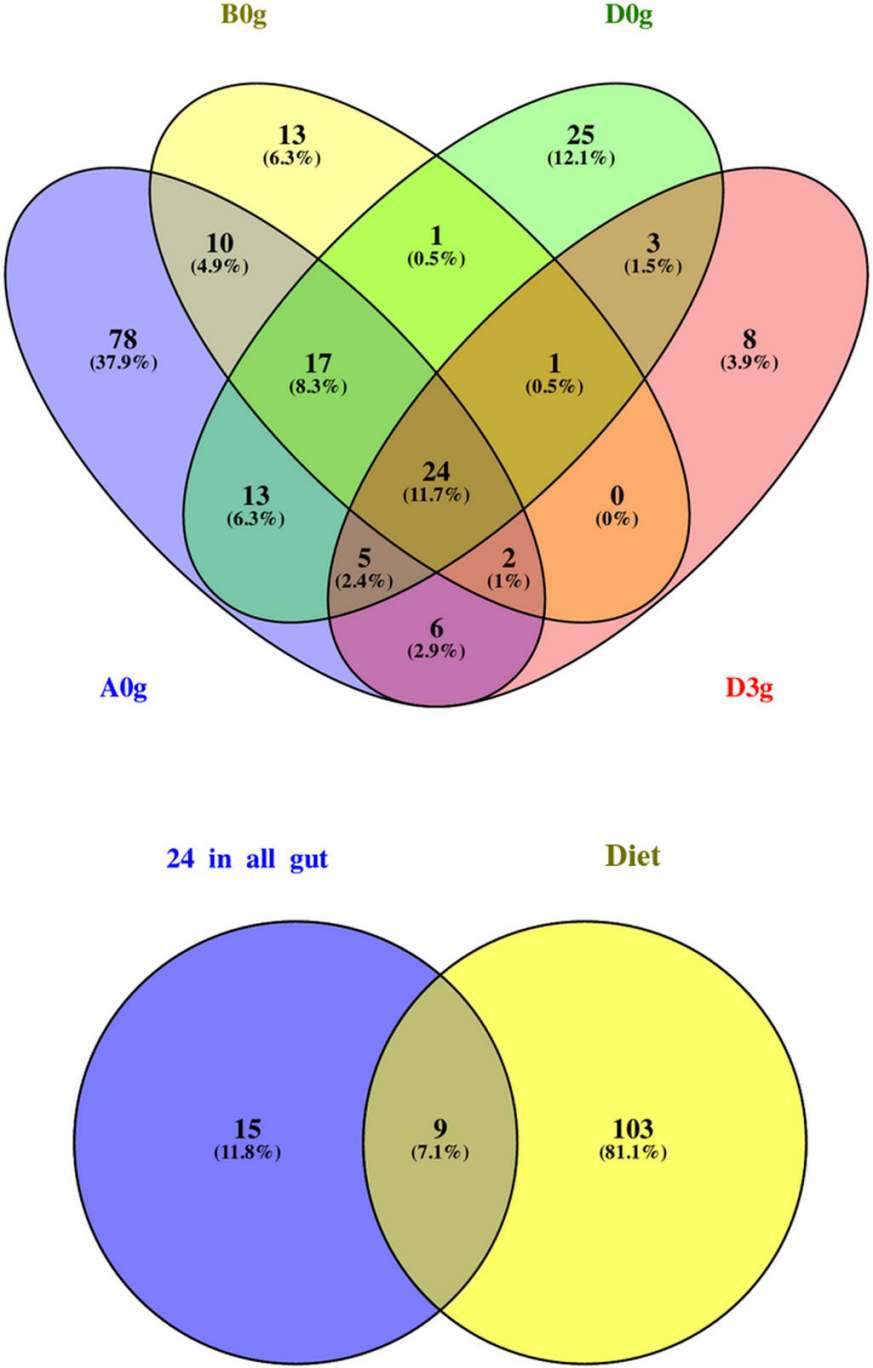
**Shared bacterial OTUs between the midguts (g) of *O. mykiss* individuals reared with three different diets (A, B, D) just before feeding at the beginning of the experiment (0) and 3 h (3) after feeding with Diet D (upper graph) along with the shared OTUs between the 24 common OTUs found in all midgut samples fed all dietary treatments (lower graph).** % indicates the contribution of each compartment to the total number of OTUs per Venn diagram.

**Fig. 2.**
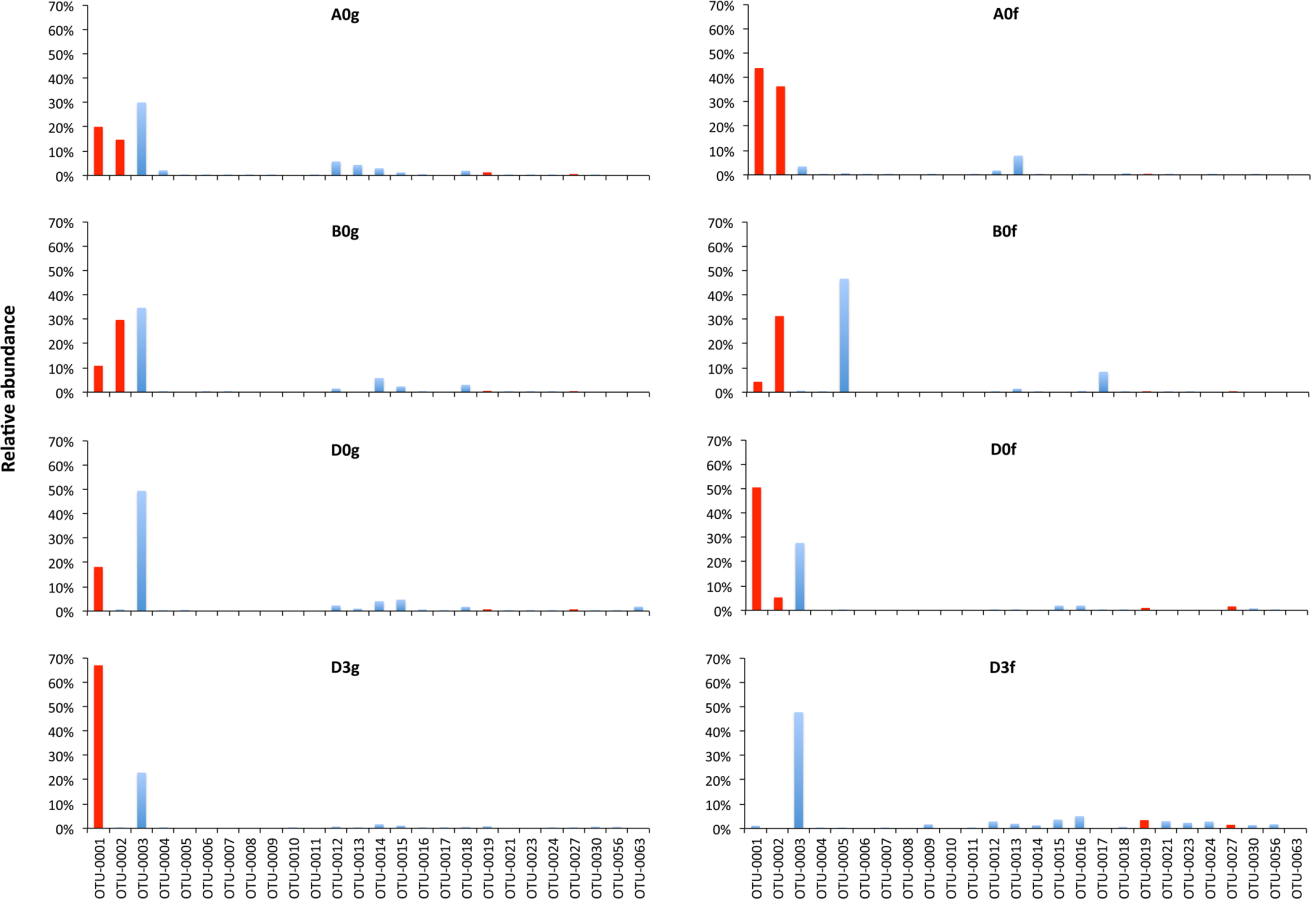
**The most abundant OTUs in the midgut (g) and faeces (f) of *O.mykiss* reared with three different diets (A,B,D) just before feeding at the beginning of the experiment (0) and 3 h (3) after feeding one meal from Diet D.** Red indicates a core OTU.

According to the Hamady and Knight (2009) ‘core microbiome’ definition, in this study core microbiota consisted of OTUs that fulfilled two prerequisites: (a) to occur in all gut samples, regardless of the supplied diet and time after feeding (A0g, B0g, D0g, D3g) and (b) to not be found in the supplied feed. Thus, the core microbiota in this study consists of 15 OTUs (Figs 1 and 2). From these OTUs, four were among the most abundant (Fig. 2; Table S2).

**Fig. 3.**
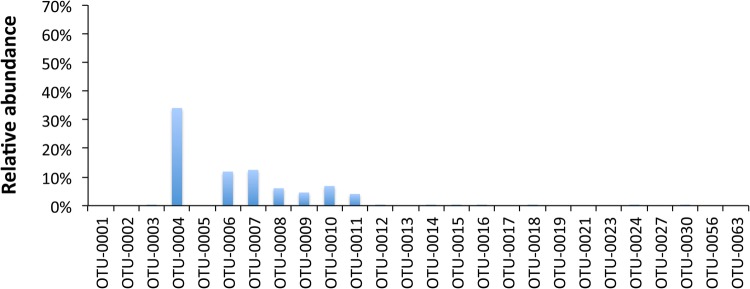
**Most abundant OTUs in reared *O. mykiss* fed Diets A, B and D.**

A total of 26 OTUs dominated across all treatments (Fig. 2). In midgut samples, three OTUs dominated (OTU-0001, -0002 and -0003). These OTUs are distantly related to *Mycoplasma* sp. (OTU-0001) and *Acetanaeromicrobium* sp. (OTU-0002) and are closely related to *Bacillus* sp. (OTU-0003) (Table S2). Regarding the faeces samples, similar dominance was observed for Diets A and D. OTU-0005 (98% similar to *Cetobacterium* sp.) dominated in the faeces of Diet B. In the pooled diet sample (Fig. 3), a cyanoabacterium-related (*Chroocidiopsis* sp.) OTU-0004 dominated, while another six OTUs (OTU-0006, -0007, -0008, -0009, -0010, -0011) appeared to have ca. 4–12% dominance; these OTUs never exceeded, if present at all, ca. 0.1% in all midgut samples or ca. 1.7% in sample D3f (Fig. 2), and for this reason are not considered important.

**Fig. 4.**
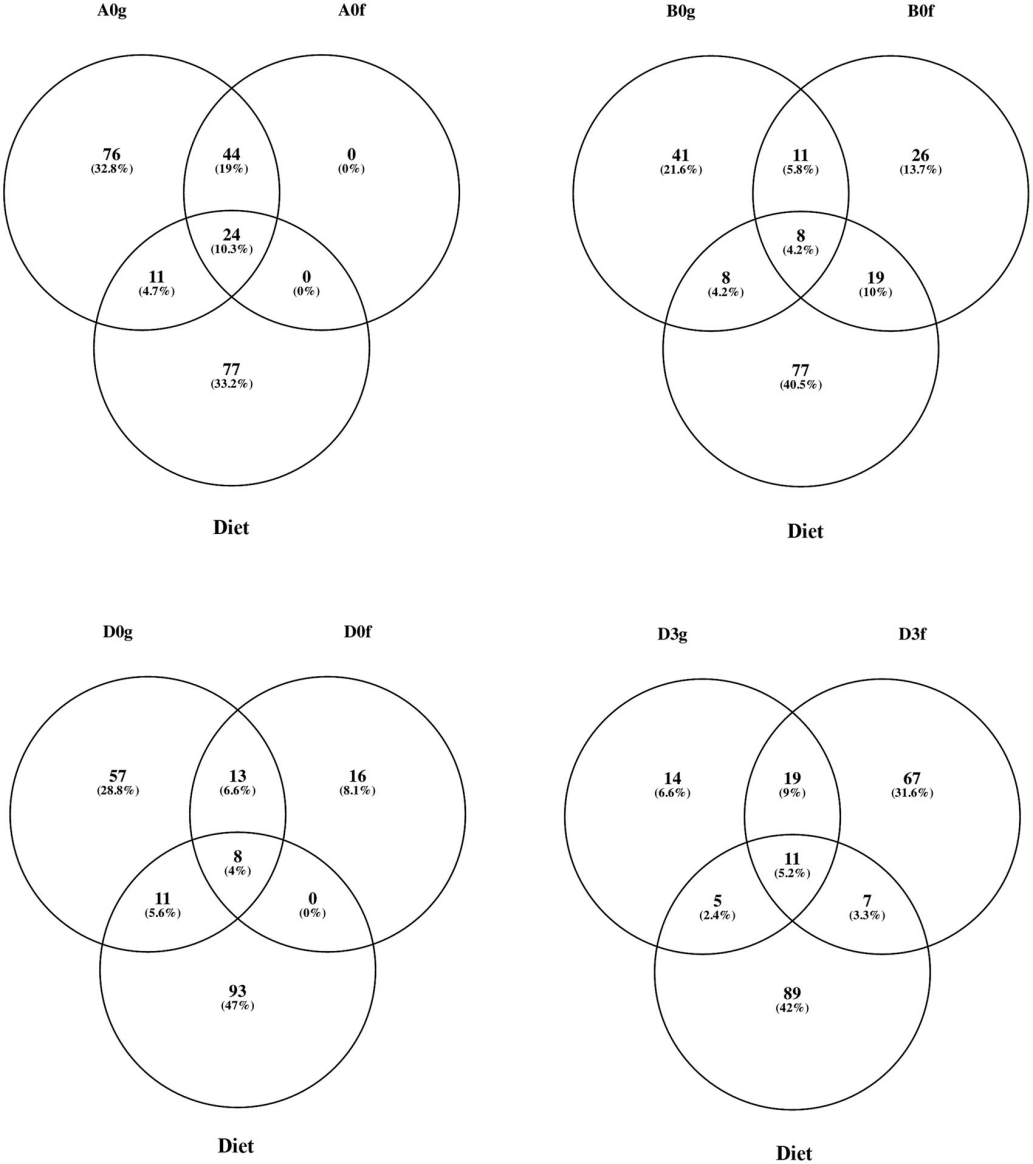
**Shared OTUs between the midgut (g), faeces (f) and composite feed of *O.mykiss* reared with three different diets (A,B,D) just before feeding at the beginning of the experiment (0) and 3 h (3) after feeding with Diet D.** % indicates the contribution of each compartment to the total number or OTUs per Venn diagram.

**Fig. 5.**
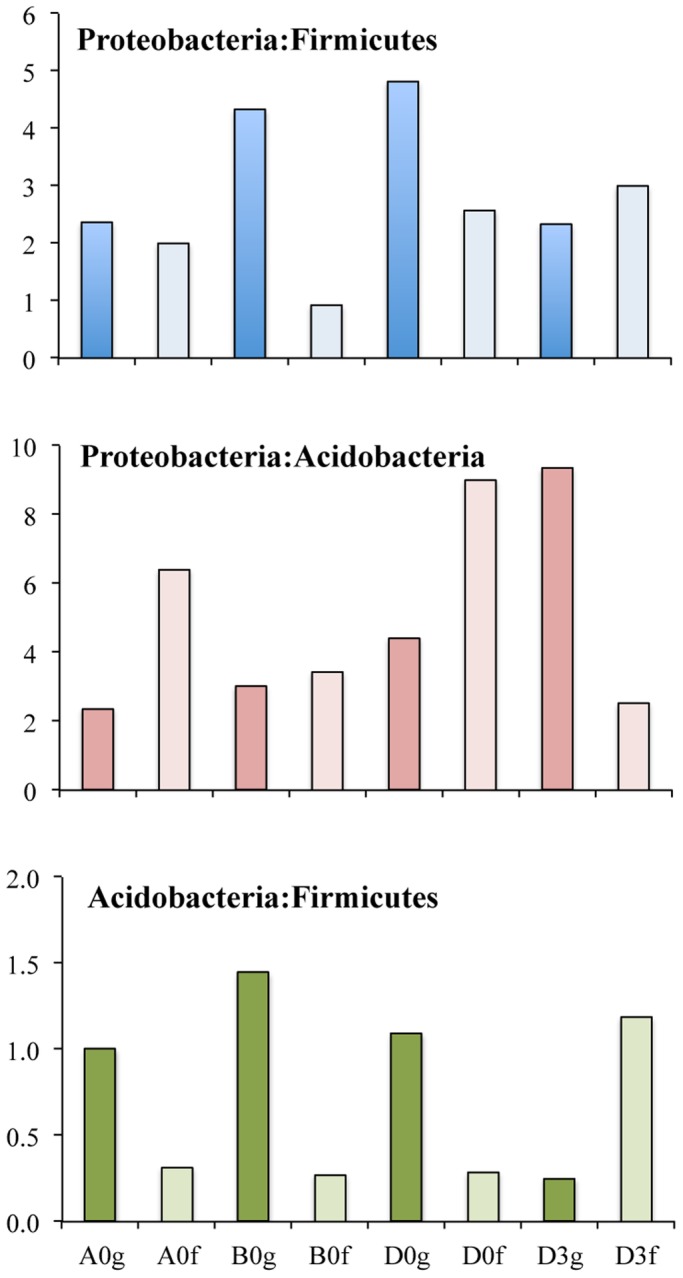
**Midgut-faeces inconsistencies based on the ratio of the number of OTUs belonging to the three most abundant bacterial phyla found in *O.mykiss* reared with three different diets (A,B,D) at the beginning of the experiment (0) and at 3 h (3) after feeding with Diet D.** Dark-coloured and light-coloured bars indicate gut and faecal samples respectively.

The shared number of OTUs between a midgut sample and its faeces within the corresponding diets, was considered a proxy in this study for the examination of the effect of different diet compositions on which bacteria were removed from the gut, i.e. ‘flushed’ (Fig. 4). The percentage of ‘flushed’ OTUs, i.e. the number of shared OTUs between the midgut and its faeces/total number of OTUs in the midgut, for Diets A, B and D at 0 h and D at 3 h after feeding were 28.4%, 16.2%, 14.6% and 38.8%, respectively. The effect of the different diets was also reflected in the midgut-faeces inconsistency defined as the ratio of the three most abundant bacterial phyla between a midgut sample and its respective faeces sample (Fig. 5). All three ratios, when investigated, i.e. Proteobacteria:Firmicutes, Proteobacteria:Acidobacteria and Acidobacteria:Firmicutes, showed a similar change at 0 h, but it was always the opposite at 3 h after feeding for Diet D.

### Short-term feeding a single meal has impact on gut microbiota

**Fig. 6.**
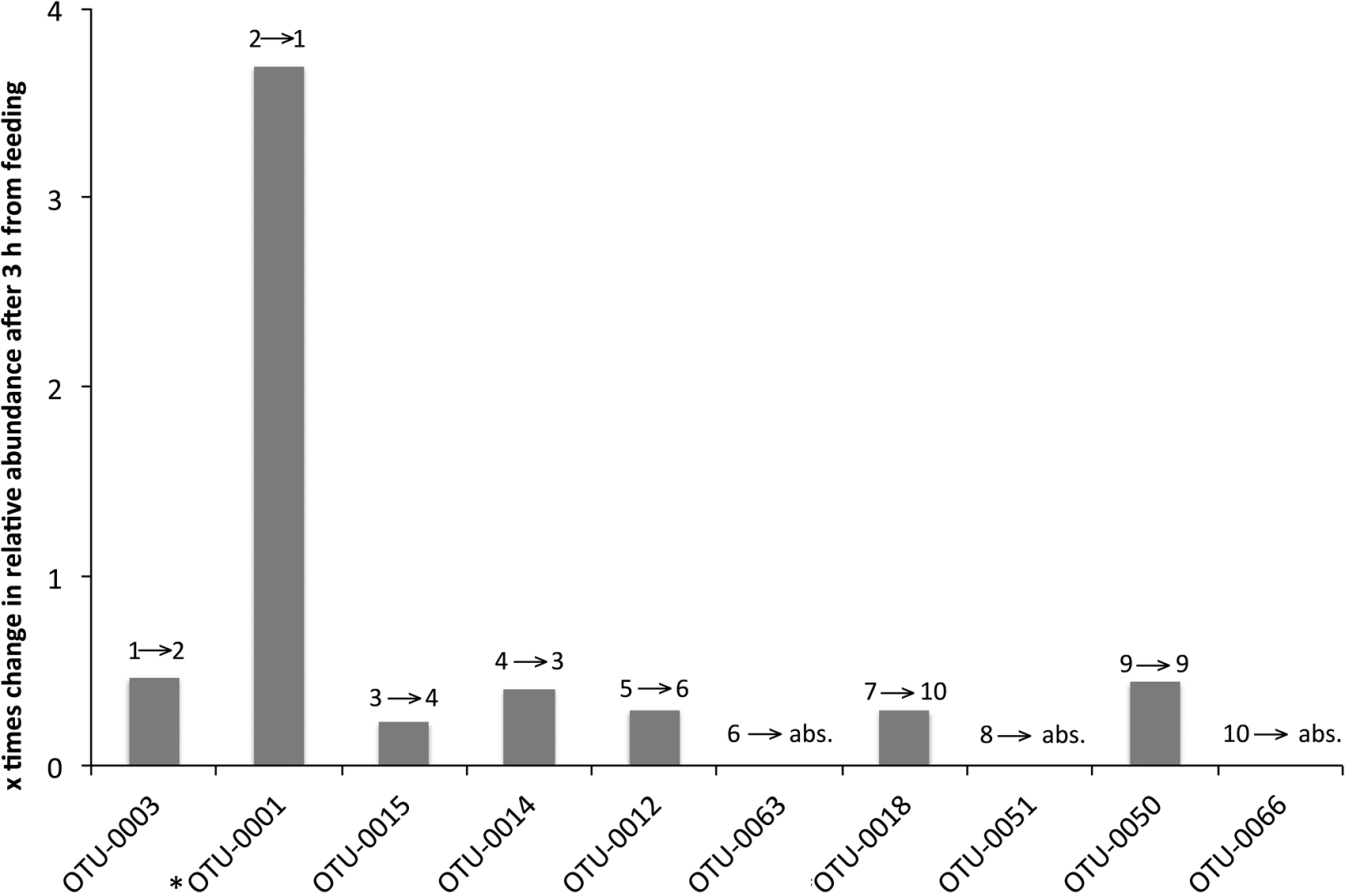
**Relative change in the abundance of the most abundant midgut OTUs of *O.mykiss* 3 h after feeding with Diet D.** Numbers above each bar indicate the change in OTU ranking from 0 h → 3 h. * indicates a core OTU; ‘abs.’ indicates absent.

With Diet D, we investigated the short-term (3 h after feeding) impact of feeding a single meal. The number of midgut OTUs decreased from 89 at 0 h (pre-feeding time) to 49 at 3 h after feeding a single meal (Table 1). Although no statistically significant differences were observed between the most abundant OTUs at 0 h and 3 h (*t*-test *P*=0.456), structural changes in the microbiota profiles were observed (Fig. 6). The relevant abundance of OTU-0003, which was the dominant OTU in the gut in Diet D at 0 h (D0g) decreased 0.5 times, while the opposite happened for OTU-0001 (increased 3.7 times). The rest of the eight most abundant OTUs at 0 h showed little decrease in their relative abundance after 3 h. The rank abundance of another five of the ten most abundant OTUs showed a slight change, while another three OTUs were not detected at all 3 h after feeding. The bacterial species richness of the diet had little overlap with both the 0 h and 3 h gut microbiota readings (Fig. S1).

### Phylogenetic affiliation of the gut microbiota

The putative phylogenetic affiliation and origin of OTUs designated as ‘core’, ‘core and most abundant’ or ‘most abundant’ is shown in Table S2. The four OTUs (OTU-0001, -0002, -0019, -0027) which belonged to the group ‘core and most abundant’ were all related to fish guts, including the rainbow trout. In addition, they were most likely related to genera which are associated with the GIT of several animals (e.g. *Mycoplasma*, *Bacillus* and *Photobacterium*; Table S2). Their putative phylogenetic affiliation falls in the Tenericutes, Firmicutes and one unaffiliated OTU. Eight out of the 11 ‘core’ OTUs were also related to various animal gut habitats. The rest of this group’s OTUs were related to other animal tissues or associated with composting processes. The Firmicutes dominated in this group as well. Finally, 22 OTUs were characterised as ‘most abundant’ but not belonging to the core microbiota. Twelve of these OTUs were animal gut related, four related to animal tissues or products and the rest to water, soil or plant material. The dominant phylum in this group was the γ-Proteobacria and mostly of the order Pseudomonadales. Other important phyla were the Firmicutes (Lactobacillales), Bacteroidetes, β-Proteobacteria, Tenericutes and Fusobacteria, while six OTUs could not be affiliated to known taxonomic groups.

## DISCUSSION

### Gut community structure

Changes in the composition of bacterial communities are due to changes in the supplied substrates and predation. Since the occurrence of bacterial grazers (mostly nanoflagellates or small ciliates) in healthy gut environments is considered low, the changes in community structures are attributed mostly to dietary supply. Protists are known to be related to the gut environment mainly at their first developmental stages when used as feed (Overton et al., 2010; Zingel et al., 2012), although in some cases these protists are not among primary food sources (de Figueiredo et al., 2012). Thus, in this study, the observed changes in rainbow trout gut bacterial communities, due to the supply of three types of diets, can be attributed mostly to the differences in the composition of these diets. Rainbow trout that were fed yeast diets had similar bacterial diversity and lower abundance of *Leuconostocaceae* and *Photobacterium* compared with those that were fed fishmeal (Huyben et al., 2018a,b).

It has been proposed that fish GIT bacterial communities can be shaped by external abiotic (e.g. salinity and temperature) as well as biotic factors (e.g. trophic level of the host and its phylogeny) for allochthonous and autochthonous bacteria (Sullam et al., 2012; Dehler et al., 2017b; Huyben et al., 2018a,b). Herein, the same cohort of individuals was used. Thus, the observed differences in trout GIT bacterial community structures were assumed to be due to the differences in the diets offered and consumed.

### Core microbiota

Several studies have demonstrated the impact of diet on gut microbiota in rainbow trout, particularly after a supply of plant ingredients in their diets (Heikkinen et al., 2006; Merrifield et al., 2009; Desai et al., 2012; Mansfield et al., 2010; Navarrete et al., 2012), showing that a set of bacteria occur in the animal's GIT irrespective of the diet offered, forming its ‘core microbiota’ (Wong et al., 2013). In our study, we identified 15 such core bacterial OTUs. This group contained both fundamental and possible keystone OTUs (*sensu* Magurran, 2004), i.e. bacteria with high relative abundance and bacteria which, although they had low relative abundance, their occurrence was not affected by the diet offered. Only four of them were among the most abundant, suggesting that the mere dominance of a bacterial OTU does not necessarily imply its role as a fundamental organism for the midgut of the rainbow trout. Despite the fact that the importance of much less dominant, common, or even rare OTUs in microbial communities is now considered of major importance for the succession and functioning of microbial communities (Sogin et al., 2006; Pedrós-Alió, 2012; Shade et al., 2014), this potential role of the rare biosphere has not attracted much attention in the GIT microbial communities of reared species.

The four most abundant core OTUs were related (Table S2) to major taxa, which include cultured representatives and phylotypes originating from the GIT of other fish gut including the rainbow trout. This reinforces the crucial role these microorganisms have for the ecophysiology of the gut. The Tenericutes (OTU-0001) have been found to have increased dominance in healthy rainbow trout (Lyons et al., 2017), while the Firmicutes (OTU-0002, -0019) are among the most common and metabolically important bacteria in the GIT of several healthy fish (Givens et al., 2015; Lowrey et al., 2015; Dehler et al., 2017a; Huyben et al., 2018a). Both the Tenericutes and the Firmicutes have also been found in the core microbiota of the Atlantic salmon parr (Dehler et al., 2017a) and the Firmicutes and Proteobacteria have been characterised as members of the rainbow trout (Wong et al., 2013) and common carp (*Cyprinus carpio* L.) (Li et al., 2013) core microbiota. The rest of the 11 core OTUs, which had low relative abundance, also belonged to the Firmicutes and the α-Proteobacteria, while some could not be affiliated to any of the known major taxa. As in the core and most abundant OTUs, all of these bacteria were related to phylotypes originating from animal GIT, or other tissues, or composting environments, implying their role in food processing in the midgut. Rainbow trout require a high protein diet (Panserat et al., 2013) and are known to have a low rate of utilisation of dietary carbohydrates when fed with a high carbohydrate diet, which is associated with decreased growth (Wilson, 1994; Polakof et al., 2012; Geurden et al., 2014). The dominant occurrence of Tenericutes and Firmicutes might assist in the nutritional processes of complex and undigested polysaccharides, as has been shown in other fish species (Ni et al., 2014), a sea urchin (Hakim et al., 2016) and mammals (Zhu et al., 2015; Yan et al., 2016) including humans (Gill et al., 2006).

Recently, it was shown that there is a direct linear relationship between the 16S rRNA gene copy number and the growth rate of bacteria (Roller et al., 2016). Firmicutes and Proteobacteria are known to have the highest 16S rRNA gene copy numbers (Kembel et al., 2012; Sun et al., 2013; Roller et al., 2016), having on average 6.0 and 5.4 copies, respectively (Sun et al., 2013), based on more than 200 species per phylum. Based on 147 publically available Tenericutes genomes (accessed 20 Nov. 2016) from the Microbial Genome Resources website (https://www.ncbi.nlm.nih.gov/genome/microbes/), we found that this taxon has on average 4.7±2.29 (median=4) 16S rRNA gene copies, which is comparable to that of Firmicutes and Proteobacteria. This, according to Roller et al. (2016), predicts high growth rates of ca. 0.64 and 0.18 for Firmicutes and Tenericutes respectively, and thus explains their dominance in the rainbow trouts’ guts that we found in this study. What metabolic pathways these bacteria are engaged in within the fish's midgut remains to be seen.

### ‘Flushed’ OTUs and midgut-faeces inconsistency

It has been assumed that gut mirobiota of healthy hosts undergoes strong environmental selection (Faust and Raes, 2016) and that such stability is reflected in the stool microbiota consistency (Bashan et al., 2016). If the diets used in our study had a similar effect on the trouts’ midgut microbiota, the gut-faeces consistency should have been the same. We observed that not only this did not happen in terms of ‘flushed’ bacterial OTUs (Fig. 4), but the ratio of the dominant phyla differed between the midgut and the faeces (Fig. 5). These observations implied that different diets can alter gut microbiota not only by selecting the dominant species, but also through variable ‘flushing’ of the gut's bacteria.

### Effect of short-term feeding a single meal on the gut bacteria

It is known that during development and change of feeding mode, fish gut microbiota undergoes significant alterations (Stephens et al., 2016). In this study, we investigated if such community shifts occur even under after a very short (3 h) time after feeding (Fig. 6). It was shown that such a short time is not sufficient to cause any significant succession, but it can cause community structural changes. These initial perturbations in species richness and the relative abundance of (at least) the dominant ones are the first step for changes, even in the metabolic diversity of a habitat (e.g. Xia et al., 2014). We observed that during the 3 h interval, the core Tenericutes-related OTU-0001 changed its relative abundance by ca. 4 times. This is an indication that this species can outcompete the others, at least with Diet D, which had the highest ratio of plant protein to oil and high marine protein to oil (Table S1). A possible explanation is that this OTU might originate from the phylum's members found in termite gut, where the Tenericutes are associated with the decomposition of the ingested plant material (Boucias et al., 2013; Tarayre et al., 2015).

## CONCLUSIONS

The fish gut microbial communities can contribute nutrients and energy to the host via the fermentation of non-digestible dietary components and maintain a balance with the fish's metabolism and immune system. Changes in diet composition can been seen as a perturbation factor for the GIT microbial communities of the host organism, causing shifts in these microbial communities. Such shifts can lead even to pathogen invasion, i.e. ‘restaurant hypothesis’ (Conway and Cohen, 2015). This study revealed such changes in the micriobiota community profile of the rainbow trout gut and faeces in response to diet and time after feeding a single meal. Whether such changes at the community level reflect different ecophysiological roles of the GIT bacterial communities remain to be elucidated.

## MATERIALS AND METHODS

### Experimental procedures and sampling

The experimental procedure was in line with the EU legal framework related to the welfare and protection of animals used for scientific purposes (Directive 2010/63/EU) and the guidelines of legislation in the UK that governs the ethical treatment of animals (Animal Scientific Procedures Act, 1986, UK). Three commercial diets were used, encoded as Diets A, B and D (Table S1). The diets were formulated with higher marine fishmeal/fish oil and lower marine fishmeal/fish oil content by a commercial company. The chemical composition of the diet was analysed for protein content using the Kjeldahl method and for lipid content using the Soxtherm method (AOAC, 1995). Dry matter was measured after drying at 105°C for 24 h.

A total of 360 juvenile rainbow trout individuals of mixed sex, weighing approximately 45.0±1.11 g, were randomly distributed to nine 250 l freshwater tanks with 40 individuals in each tank, corresponding to the three different dietary treatments (A, B and D). The rearing conditions were as described in Mente et al. (2017). Briefly, temperature was set at 12°C, at pH 7.6 under natural photoperiod with 90% oxygen saturation. Fish were fed the experimental diets *ad libitum,* by hand, twice daily at 09:00 and 15:00 for 5 weeks. The fish were acclimatised in the tanks for 2 weeks prior to the beginning of the experimental feeding with the Diets A, B and D. At the end of the growth experiment, fish were fasted for 24 h. From the total 18 fish that were sampled, using two from each tank, for this study, three healthy fish from each dietary treatment were removed and euthanised (S1K method) by anaesthesia (phenoxyethanol) followed by destruction of the brain. Their gut tissues were immediately removed, their midgut tissue was dissected out with sterile instruments, which were frozen in liquid nitrogen and used as the pre-feeding time (t)=0 h group for analysis of the gut microbiota. The remaining trout in the dietary treatment D were fed normally with Diet D. Diet D was selected for the short-term feeding a single meal experiment since it was the diet with the higher plant protein/lipid and high marine protein/lipid level. A further three fish, one from each experimental tank, were randomly selected 3 h after feeding Diet D. They were euthanised, their gut tissue was removed and the midgut tissue was rapidly dissected out as described above. To enrich the resident microorganisms (i.e. occurring on or inside the gut tissue) and not those associated with the digesta (i.e. transient), gut material was removed by applying gentle mechanical force using flat forceps. The remaining midgut tissue was sequentially rinsed three times in sterile particle-free (<0.2 μm) distilled water (SPFDW) as described in Kormas et al. (2014), then frozen in liquid nitrogen and kept at −80°C until analysis.

Faeces were also manually collected from different fish at the pre-feeding (t=0 h) time from all tanks and dietary treatments and 3 h (t=3 h) after feeding for Diet D. Faeces were rinsed in SPFDW immediately after collection and kept at −80°C until further analysis. The time between gut or faeces sampling and storage never exceeded 4 h. Finally, a triplicated sample of all the three diets, consisting of 1 g from each diet, was also collected and stored at −80°C until further analysis.

### Molecular and sequencing analysis

DNA was extracted from each individual 200 mg sample by using the PowerMax Soil DNA Isolation kit (MoBio, Carlsbad, CA, USA) according to manufacturer's protocol. The 454 FLX titanium tag pyrosequencing platform (Roche, Brandford, CT, USA) was used, targeting the V3–V4 region (Martínez-Porchas et al., 2016) of the 16S rRNA gene by using the primer pair S-DBact-0341-b-S-17 (5′-CCTACGGGNGGCWGCAG-3′) and S-D-Bact-0785-a-A-21 (5′-GACTACHVGGGTATCTAATCC-3′) (Klindworth et al., 2012) according to Dowd et al. (2008) at the MRDNA Ltd. (Shallowater, TX, USA) sequencing facilities. In brief, a one-step 30 cycle Polymerase Chain Reaction (PCR) was applied using HotStarTaq Plus Master Mix Kit (Qiagen) with the same amount of DNA template (ca. 8-10 ng/μl). PCR conditions included: 94°C for 3 min, then 28 cycles at 94°C for 30 s; 53°C for 40 s and 72°C for 1 min; and a final elongation step at 72°C for 5 min. All resulting data were processed with MOTHUR software (v. 1.36.0) (Schloss et al., 2009). Quality control of data analysis included flowgrams denoising by PyroNoise software (Quince et al., 2009), keeping only the sequences with ≥250 bp with no homopolymers of ≥8 bp. The remaining sequences were aligned in the SILVA 119 database (Pruesse et al., 2007). The sequences were binned into OTUs and were clustered based on the average neighbour algorithm with 97% similarity as the cut-off level (Kunin et al., 2010; Stackebrandt and Goebel, 1994). The unique OTUs were taxonomically classified by using the SILVA 119 database (Pruesse et al., 2007). The batch of sequences from this study can be accessed at the Short Reads Archive (http://www.ncbi.nlm.nih.gov/sra) with accession number SAMN04028037.

Differences in the growth performance of the fish (initial and final weights) were analysed using one-way ANOVA followed by Tukey’s multiple-range test. Homogeneity was confirmed using Levene's test. All statistical analyses were carried out using SPSS Statistics, version 22 (IBM).

## Supplementary Material

Supplementary information

## References

[BIO034397C1] AOAC (1995). *Official Methods of Analysis of AOAC International*. Washington: Association of Official Analytical Chemists.

[BIO034397C2] Bashan A., Gibson T. E., Friedman J., Carey V. J., Weiss S. T., Hohmann E. L. and Liu Y.-Y. (2016). Universality of human microbial dynamics. *Nature* 534, 259-262. 10.1038/nature18301 27279224 PMC4902290

[BIO034397C3] Berg R. D. (1996). The indigenous gastrointestinal microflora. *Trends Microbiol.* 4, 430-435. 10.1016/0966-842X(96)10057-3 8950812

[BIO034397C4] Besemer K., Peter H., Logue J. B., Langenheder S., Lindstrom E. S., Tranvik L. J. and Battin T. J. (2012). Unraveling assembly of stream biofilm communities. *ISME J.* 6, 1459-1468. 10.1038/ismej.2011.205 22237539 PMC3400417

[BIO034397C5] Boucias D. G., Cai Y., Sun Y., Lietze V.-U., Sen R., Raychoudhury R. and Scharf M. E. (2013). The hindgut lumen prokaryotic microbiota of the termite Reticulitermes flavipes and its responses to dietary lignocellulose composition. *Mol. Ecol.* 22, 1836-1853. 10.1111/mec.12230 23379767

[BIO034397C6] Cahill M. M. (1990). Bacterial flora of fishes: a review. *Microb. Ecol.* 19, 21-41. 10.1007/BF02015051 24196252

[BIO034397C7] Carter C. G., Houlihan D. F., Kiessling A., Medale F. and Jobling M. (2001). Physiological effects of feeding. In *Food Intake in Fishes* (ed. Houlihan D. F., Boujard T. and Jobling M.), pp. 297-331. Oxford: Blackwell Scientific.

[BIO034397C8] Clements K. D., Angert E. R., Montgomery W. L. and Choat J. H. (2014). Intestinal microbiota in fishes: what's known and what's not. *Mol. Ecol.* 23, 1891-1898. 10.1111/mec.12699 24612310

[BIO034397C9] Conway T. and Cohen P. (2015). Applying the restaurant hypothesis to intestinal microbiota. *Microbe Magazine* 10, 324-328. 10.1128/microbe.10.324.1

[BIO034397C10] Coyte K. Z., Schluter J. and Foster K. R. (2015). The ecology of the microbiome: Networks, competition, and stability. *Science* 350, 663-666. 10.1126/science.aad2602 26542567

[BIO034397C11] David L. A., Maurice C. F., Carmody R. N., Gootenberg D. B., Button J. E., Wolfe B. E., Ling A. V., Devlin A. S., Varma Y., Fischbach M. A. et al. (2014). Diet rapidly and reproducibly alters the human gut microbiome. *Nature* 505, 559-563. 10.1038/nature12820 24336217 PMC3957428

[BIO034397C12] de Figueiredo D., Ferreira R., Cerqueira M. R., de Melo T., Pereira M. R., Castro B. and Correia A. N. (2012). Impact of water quality on bacterioplankton assemblage along Cétima River Basin (central western Portugal) assessed by PCR-DGGE and multivariate analysis. *Environ. Monit. Assess.* 184, 471-485. 10.1007/s10661-011-1981-2 21431313

[BIO034397C13] Dehler C. E., Secombes C. J. and Martin S. A. M. (2017a). Environmental and physiological factors shape the gut microbiota of Atlantic salmon parr (*Salmo salar* L.). *Aquaculture* 467, 149-157. 10.1016/j.aquaculture.2016.07.017 28111483 PMC5142738

[BIO034397C14] Dehler C. E., Secombes C. J. and Martin S. A. M. (2017b). Seawater transfer alters the intestinal microbiota profiles of Atlantic salmon (*Salmo salar* L.). *Sci. Rep.* 7, 13877 10.1038/s41598-017-13249-8 29066818 PMC5654775

[BIO034397C15] Desai A. R., Links M. G., Collins S. A., Mansfield G. S., Drew M. D., Van Kessel A. G. and Hill J. E. (2012). Effects of plant-based diets on the distal gut microbiome of rainbow trout (Oncorhynchus mykiss). *Aquaculture* 350-353, 134-142. 10.1016/j.aquaculture.2012.04.005

[BIO034397C16] Dowd S., Callaway T., Wolcott R., Sun Y., McKeehan T., Hagevoort R. and Edrington T. (2008). Evaluation of the bacterial diversity in the feces of cattle using 16S rDNA bacterial tag-encoded FLX amplicon pyrosequencing (bTEFAP). *BMC Microbiol.* 8, 125 10.1186/1471-2180-8-125 18652685 PMC2515157

[BIO034397C17] Faust K. and Raes J. (2016). Host-microbe interaction: rules of the game for microbiota. *Nature* 534, 182-183. 10.1038/534182a 27279206

[BIO034397C18] Findley K., Williams D. R., Grice E. A. and Bonham V. L. (2016). Health disparities and the microbiome. *Trends Microbiol.* 24, 847-850. 10.1016/j.tim.2016.08.001 27712950 PMC5695575

[BIO034397C19] Flint H. J., Duncan S. H., Scott K. P. and Louis P. (2007). Interactions and competition within the microbial community of the human colon: links between diet and health: minireview. *Environ. Microbiol.* 9, 1101-1111. 10.1111/j.1462-2920.2007.01281.x 17472627

[BIO034397C20] Geurden I., Mennigen J., Plagnes-Juan E., Veron V., Cerezo T., Mazurais D., Zambonino-Infante J., Gatesoupe J., Skiba-Cassy S. and Panserat S. (2014). High or low dietary carbohydrate:protein ratios during first-feeding affect glucose metabolism and intestinal microbiota in juvenile rainbow trout. *J. Exp. Biol.* 217, 3396-3406. 10.1242/jeb.106062 25274323

[BIO034397C21] Gill S. R., Pop M., DeBoy R. T., Eckburg P. B., Turnbaugh P. J., Samuel B. S., Gordon J. I., Relman D. A., Fraser-Liggett C. M. and Nelson K. E. (2006). Metagenomic analysis of the human distal gut microbiome. *Science* 312, 1355-1359. 10.1126/science.1124234 16741115 PMC3027896

[BIO034397C22] Givens C., Ransom B., Bano N. and Hollibaugh J. (2015). Comparison of the gut microbiomes of 12 bony fish and 3 shark species. *Mar. Ecol. Prog. Ser.* 518, 209-223. 10.3354/meps11034

[BIO034397C23] González C. J., López-Díaz T. M., García-López M. L., Prieto M. and Otero A. (1999). Bacterial microflora of wild brown trout (*Salmo trutta*), wild pike (*Esox lucius*), and aquacultured rainbow trout (*Oncorhynchus mykiss*). *J. Food Prot.* 62, 1270-1277. 10.4315/0362-028X-62.11.1270 10571316

[BIO034397C24] Hakim J. A., Koo H., Kumar R., Lefkowitz E. J., Morrow C. D., Powell M. L., Watts S. A. and Bej A. K. (2016). The gut microbiome of the sea urchin, Lytechinus variegatus, from its natural habitat demonstrates selective attributes of microbial taxa and predictive metabolic profiles. *FEMS Microbiol. Ecol.* 92, fiw146 10.1093/femsec/fiw146 27368709 PMC5975844

[BIO034397C25] Hamady M. and Knight R. (2009). Microbial community profiling for human microbiome projects: Tools, techniques, and challenges. *Genome Res.* 19, 1141-1152. 10.1101/gr.085464.108 19383763 PMC3776646

[BIO034397C26] Heikkinen J., Vielma J., Kemiläinen O., Tiirola M., Eskelinen P., Kiuru T., Navia-Paldanius D. and von Wright A. (2006). Effects of soybean meal based diet on growth performance, gut histopathology and intestinal microbiota of juvenile rainbow trout (Oncorhynchus mykiss). *Aquaculture* 261, 259-268. 10.1016/j.aquaculture.2006.07.012

[BIO034397C27] Holben W. E., Williams P., Saarinen M., Särkilahti L. K. and Apajalahti J. H. A. (2002). Phylogenetic analysis of intestinal microflora indicates a novel *Mycoplasma* phylotype in farmed and wild salmon. *Microb. Ecol.* 44, 175-185. 10.1007/s00248-002-1011-6 12082453

[BIO034397C28] Houlihan D. F., Mathers E. and Foster A. R. (1993). Biochemical correlates of growth rate in fish. In *Fish Ecophysiology* (ed. Rankin J. C. and Jensen F. B.), pp. 45-70. London: Chapman and Hall.

[BIO034397C29] Hovda M. B., Lunestad B. T., Fontanillas R. and Rosnes J. T. (2007). Molecular characterisation of the intestinal microbiota of farmed Atlantic salmon (*Salmo salar* L.). *Aquaculture* 272, 581-588. 10.1016/j.aquaculture.2007.08.045

[BIO034397C30] Huyben D., Sun L., Moccia R., Kiessling A., Dicksved J. and Lundh T. (2018a). Dietary live yeast and increased water temperature influence the gut microbiota of rainbow trout. *J. Appl. Microbiol.* 124, 1377-1392. 10.1111/jam.13738 29464844

[BIO034397C31] Huyben D., Vidaković A., Langeland M., Nyman A., Lundh T. and Kiessling A. (2018b). Effects of dietary yeast inclusion and acute stress on postprandial plasma free amino acid profiles of dorsal aorta-cannulated rainbow trout. *Aquac. Nutr.* 24, 236-246. 10.1111/anu.12551 PMC5374170 27677483

[BIO034397C32] Ingerslev H. C., von Gersdorff Jørgensen L., Lenz Strube M., Larsen N., Dalsgaard I., Boye M. and Madsen L. (2014). The development of the gut microbiota in rainbow trout (*Oncorhynchus mykiss*) is affected by first feeding and diet type. *Aquaculture* 424-425, 24-34. 10.1016/j.aquaculture.2013.12.032

[BIO034397C33] Kembel S. W., Wu M., Eisen J. A. and Green J. L. (2012). Incorporating 16S gene copy number information improves estimates of microbial diversity and abundance. *PLoS Comput. Biol.* 8, e1002743 10.1371/journal.pcbi.1002743 23133348 PMC3486904

[BIO034397C34] Kim D. H., Brunt J. and Austin B. (2007). Microbial diversity of intestinal contents and mucus in rainbow trout (Oncorhynchus mykiss). *J. Appl. Microbiol.* 102, 1654-1664. 10.1111/j.1365-2672.2006.03185.x 17578431

[BIO034397C35] Klindworth A., Pruesse E., Schweer T., Peplies J. R., Quast C., Horn M. and Glöckner F. O. (2012). Evaluation of general 16S ribosomal RNA gene PCR primers for classical and next-generation sequencing-based diversity studies. *Nucleic Acids Res.* 41, e1 10.1093/nar/gks808 22933715 PMC3592464

[BIO034397C36] Konopka A., Lindemann S. and Fredrickson J. (2015). Dynamics in microbial communities: unraveling mechanisms to identify principles. *ISME J.* 9, 1488-1495. 10.1038/ismej.2014.251 25526370 PMC4478703

[BIO034397C37] Kormas K. A., Meziti A., Mente E. and Frentzos A. (2014). Dietary differences are reflected on the gut prokaryotic community structure of wild and commercially reared sea bream (*Sparus aurata*). *Microbiol. Open* 3, 718-728. 10.1002/mbo3.202 PMC4234263 25066034

[BIO034397C38] Kunin V., Engelbrektson A., Ochman H. and Hugenholtz P. (2010). Wrinkles in the rare biosphere: pyrosequencing errors can lead to artificial inflation of diversity estimates. *Environ. Microbiol.* 12, 118-123. 10.1111/j.1462-2920.2009.02051.x 19725865

[BIO034397C39] Li X., Yan Q., Xie S., Hu W., Yu Y. and Hu Z. (2013). Gut microbiota contributes to the growth of fast-growing transgenic common carp (*Cyprinus carpio* L.). *PLoS ONE* 8, e64577 10.1371/journal.pone.0064577 23741344 PMC3669304

[BIO034397C40] Li D., Wang P., Wang P., Hu X. and Chen F. (2016). The gut microbiota: A treasure for human health. *Biotechnol. Adv.* 34, 1210-1224. 10.1016/j.biotechadv.2016.08.003 27592384

[BIO034397C41] Lowrey L., Woodhams D. C., Tacchi L. and Salinas I. (2015). Topographical mapping of the rainbow trout (*Oncorhynchus mykiss*) microbiome reveals a diverse bacterial community with antifungal properties in the skin. *Appl. Environ. Microbiol.* 81, 6915-6925. 10.1128/AEM.01826-15 26209676 PMC4561705

[BIO034397C42] Lozupone C. A., Stombaugh J. I., Gordon J. I., Jansson J. K. and Knight R. (2012). Diversity, stability and resilience of the human gut microbiota. *Nature* 489, 220-230. 10.1038/nature11550 22972295 PMC3577372

[BIO034397C43] Lyons P. P., Turnbull J. F., Dawson K. A. and Crumlish M. (2017). Phylogenetic and functional characterization of the distal intestinal microbiome of rainbow trout Oncorhynchus mykiss from both farm and aquarium settings. *J. Appl. Microbiol.* 122, 347-363. 10.1111/jam.13347 27860093

[BIO034397C44] Magurran A. E. (2004). *Measuring Biological Diversity*. Malden: Blackwell Publishing.

[BIO034397C45] Mansfield G. S., Desai A. R., Nilson S. A., Van Kessel A. G., Drew M. D. and Hill J. E. (2010). Characterization of rainbow trout (Oncorhynchus mykiss) intestinal microbiota and inflammatory marker gene expression in a recirculating aquaculture system. *Aquaculture* 307, 95-104. 10.1016/j.aquaculture.2010.07.014

[BIO034397C46] Martínez-Porchas M., Villalpando-Canchola E. and Vargas-Albores F. (2016). Significant loss of sensitivity and specificity in the taxonomic classification occurs when short 16S rRNA gene sequences are used. *Heliyon* 2, e00170 10.1016/j.heliyon.2016.e00170 27699286 PMC5037269

[BIO034397C47] Mente E., Pierce G. J., Antonopoulou E., Stead D. and Martin S. A. M. (2017). Postprandial hepatic protein expression in trout *Oncorhynchus mykiss* a proteomics examination. *Biochem. Biophys. Rep.* 9, 79-85. 10.1016/j.bbrep.2016.10.012 28955992 PMC5614473

[BIO034397C48] Merrifield D. L., Burnard D., Bradley G., Davies S. J. and Baker R. T. M. (2009). Microbial community diversity associated with the intestinal mucosa of farmed rainbow trout (Oncoryhnchus mykiss Walbaum). *Aquac. Res.* 40, 1064-1072. 10.1111/j.1365-2109.2009.02200.x

[BIO034397C49] Michl S. C., Ratten J.-M., Beyer M., Hasler M., LaRoche J. and Schulz C. (2017). The malleable gut microbiome of juvenile rainbow trout (*Oncorhynchus mykiss*): Diet-dependent shifts of bacterial community structures. *PLoS ONE* 12, e0177735 10.1371/journal.pone.0177735 28498878 PMC5428975

[BIO034397C50] Mu C., Yang Y. and Zhu W. (2016). Gut microbiota: the brain peacekeeper. *Front. Microbiol.* 7, 345 10.3389/fmicb.2016.00345 27014255 PMC4794499

[BIO034397C51] Navarrete P., Magne F., Araneda C., Fuentes P., Barros L., Opazo R., Espejo R. and Romero J. (2012). PCR-TTGE analysis of 16S rRNA from rainbow trout (*Oncorhynchus mykiss*) gut microbiota reveals host-specific communities of active bacteria. *PLoS ONE* 7, e31335 10.1371/journal.pone.0031335 22393360 PMC3290605

[BIO034397C52] Ni J., Yan Q., Yu Y. and Zhang T. (2014). Factors influencing the grass carp gut microbiome and its effect on metabolism. *FEMS Microbiol. Ecol.* 87, 704-714. 10.1111/1574-6941.12256 24256454

[BIO034397C53] Nyman A., Huyben D., Lundh T. and Dicksved J. (2017). Effects of microbe- and mussel-based diets on the gut microbiota in Arctic charr (*Salvelinus alpinus*). *Aquac. Rep.* 5, 34-40. 10.1016/j.aqrep.2016.12.003

[BIO034397C54] Overton J. L., Meyer S., Støttrup J. G. and Peck M. A. (2010). Role of heterotrophic protists in first feeding by cod (Gadus morhua) larvae. *Mar. Ecol. Prog. Ser.* 410, 197-204. 10.3354/meps08658

[BIO034397C55] Panserat S., Kaushik S. and Médale F. (2013). Rainbow trout as a model for nutrition and nutrient metabolism studies. In *Trout: From Physiology to Conservation* (ed. Polakof S., Moon T. W.), pp. 131-153. Hauppauge: Nova Publishers.

[BIO034397C56] Pedrós-Alió C. (2012). The rare bacterial biosphere. *Ann. Rev. Mar. Sci.* 4, 449-466. 10.1146/annurev-marine-120710-100948 22457983

[BIO034397C57] Polakof S., Panserat S., Soengas J. L. and Moon T. W. (2012). Glucose metabolism in fish: a review. *J. Comp. Physiol. B* 182, 1015-1045. 10.1007/s00360-012-0658-7 22476584

[BIO034397C58] Pond M. J., Stone D. M. and Alderman D. J. (2006). Comparison of conventional and molecular techniques to investigate the intestinal microflora of rainbow trout (*Oncorhynchus mykiss*). *Aquaculture* 261, 194-203. 10.1016/j.aquaculture.2006.06.037

[BIO034397C59] Pruesse E., Quast C., Knittel K., Fuchs B., Ludwig W., Peplies J. and Glöckner F. (2007). SILVA: a comprehensive online resource for quality checked and aligned ribosomal RNA sequence data compatible with ARB. *Nucleic Acids Res.* 35, 7188-7196. 10.1093/nar/gkm864 17947321 PMC2175337

[BIO034397C60] Quince C., Lanzen A., Curtis T. P., Davenport R. J., Hall N., Head I. M., Read L. F. and Sloan W. T. (2009). Accurate determination of microbial diversity from 454 pyrosequencing data. *Nat. Methods* 6, 639-641. 10.1038/nmeth.1361 19668203

[BIO034397C61] Ringø E. and Olsen R. E. (1999). The effect of diet on aerobic bacterial flora associated with intestine of Arctic charr (*Salvelinus alpinus* L.). *J. Appl. Microbiol.* 86, 22-28. 10.1046/j.1365-2672.1999.00631.x 10030010

[BIO034397C62] Ringø E., Zhou Z., Vecino J. L. G., Wadsworth S., Romero J., Krogdahl Å., Olsen R. E., Dimitroglou A., Foey A., Davies S. et al. (2016). Effect of dietary components on the gut microbiota of aquatic animals. A never-ending story? *Aquac. Nutr.* 22, 219-282. 10.1111/anu.12346

[BIO034397C63] Roller B. R. K., Stoddard S. F. and Schmidt T. M. (2016). Exploiting rRNA operon copy number to investigate bacterial reproductive strategies. *Nat. Microbiol.* 1, 16160 10.1038/nmicrobiol.2016.160 27617693 PMC5061577

[BIO034397C64] Schloss P. D., Westcott S. L., Ryabin T., Hall J. R., Hartmann M., Hollister E. B., Lesniewski R. A., Oakley B. B., Parks D. H., Robinson C. J. et al. (2009). Introducing mothur: open-source, platform-independent, community-supported software for describing and comparing microbial communities. *Appl. Environ. Microbiol.* 75, 7537-7541. 10.1128/AEM.01541-09 19801464 PMC2786419

[BIO034397C65] Shade A., Jones S. E., Caporaso J. G., Handelsman J., Knight R., Fierer N. and Gilbert J. A. (2014). Conditionally rare taxa disproportionately contribute to temporal changes in microbial diversity. *mBio* 5, e01371-e01314. 10.1128/mBio.01371-14 25028427 PMC4161262

[BIO034397C66] Sogin M. L., Morrison H. G., Huber J. A., Welch D. M., Huse S. M., Neal P. R., Arrieta J. M. and Herndl G. J. (2006). Microbial diversity in the deep sea and the underexplored “rare biosphere”. *Proc. Natl. Acad. Sci. USA* 103, 12115-12120. 10.1073/pnas.0605127103 16880384 PMC1524930

[BIO034397C67] Spanggaard B., Huber I., Nielsen J., Nielsen T., Appel K. F. and Gram L. (2000). The microflora of rainbow trout intestine: a comparison of traditional and molecular identification. *Aquaculture* 182, 1-15. 10.1016/S0044-8486(99)00250-1

[BIO034397C68] Stackebrandt E. and Goebel B. M. (1994). Taxonomic note: a place for DNA:DNA reassociation and 16S rRNA sequence analysis in the present species definition in bacteria. *Int. J. Syst. Bacteriol.* 44, 846-849. 10.1099/00207713-44-2-265

[BIO034397C69] Stephens W. Z., Burns A. R., Stagaman K., Wong S., Rawls J. F., Guillemin K. and Bohannan B. J. M. (2016). The composition of the zebrafish intestinal microbial community varies across development. *ISME J.* 10, 644-654. 10.1038/ismej.2015.140 26339860 PMC4817687

[BIO034397C70] Sullam K. E., Essinger S. D., Lozupone C. A., O'Connor M. P., Rosen G. L., Knight R. O. B., Kilham S. S. and Russell J. A. (2012). Environmental and ecological factors that shape the gut bacterial communities of fish: a meta-analysis. *Mol. Ecol.* 21, 3363-3378. 10.1111/j.1365-294X.2012.05552.x 22486918 PMC3882143

[BIO034397C71] Sun D.-L., Jiang X., Wu Q. L. and Zhou N.-Y. (2013). Intragenomic heterogeneity of 16s rrna genes causes overestimation of prokaryotic diversity. *Appl. Environ. Microbiol.* 79, 5962-5969. 10.1128/AEM.01282-13 23872556 PMC3811346

[BIO034397C72] Tarayre C., Bauwens J., Mattéotti C., Brasseur C., Millet C., Massart S., Destain J., Vandenbol M., De Pauw E., Haubruge E. et al. (2015). Multiple analyses of microbial communities applied to the gut of the wood-feeding termite *Reticulitermes flavipes* fed on artificial diets. *Symbiosis* 65, 143-155. 10.1007/s13199-015-0328-0

[BIO034397C73] Widder S., Allen R. J., Pfeiffer T., Curtis T. P., Wiuf C., Sloan W. T., Cordero O. X., Brown S. P., Momeni B., Shou W. et al. (2016). Challenges in microbial ecology: building predictive understanding of community function and dynamics. *ISME J.* 10, 2557-2568. 10.1038/ismej.2016.45 27022995 PMC5113837

[BIO034397C74] Wilson R. P. (1994). Utilization of dietary carbohydrate by fish. *Aquaculture* 124, 67-80. 10.1016/0044-8486(94)90363-8

[BIO034397C75] Wong S., Waldrop T., Summerfelt S., Davidson J., Barrows F., Kenney P. B., Welch T., Wiens G. D., Snekvik K., Rawls J. F. et al. (2013). Aquacultured rainbow trout (*Oncorhynchus mykiss*) possess a large core intestinal microbiota that is resistant to variation in diet and rearing density. *Appl. Environ. Microbiol.* 79, 4974-4984. 10.1128/AEM.00924-13 23770898 PMC3754725

[BIO034397C76] Xia J. H., Lin G., Fu G. H., Wan Z. Y., Lee M., Wang L., Liu X. J. and Yue G. H. (2014). The intestinal microbiome of fish under starvation. *BMC Genomics* 15, 1-11. 10.1186/1471-2164-15-266 24708260 PMC4234480

[BIO034397C77] Yan X., Feng B., Li P., Tang Z. and Wang L. (2016). Microflora disturbance during progression of glucose intolerance and effect of sitagliptin: an animal study. *J. Diabetes Res.* 2016, 2093171 10.1155/2016/2093171 27631013 PMC5007364

[BIO034397C78] Zarkasi K. Z., Taylor R. S., Glencross B. D., Abell G. C. J., Tamplin M. L. and Bowman J. P. (2017). In vitro characteristics of an Atlantic salmon (*Salmo salar* L.) hind gut microbial community in relation to different dietary treatments. *Res. Microbiol.* 168, 751-759. 10.1016/j.resmic.2017.07.003 28728852

[BIO034397C79] Zhu Y., Wang C. and Li F. (2015). Impact of dietary fiber/starch ratio in shaping caecal microbiota in rabbits. *Can. J. Microbiol.* 61, 771-784. 10.1139/cjm-2015-0201 26361938

[BIO034397C80] Zingel P., Paaver T., Karus K., Agasild H. and Nõges T. (2012). Ciliates as the crucial food source of larval fish in a shallow eutrophic lake. *Limnol. Oceanogr.* 57, 1049-1056. 10.4319/lo.2012.57.4.1049

