# Supplementary material for: Core versus diet-associated and postprandial bacterial communities of the rainbow trout (Oncorhynchus mykiss) midgut and faeces
Source: Biol Open. 2018 May 18;7(6):bio034397. doi: 10.1242/bio.034397 (PMC6031335; doi:10.1242/bio.034397)
Supplement: Supplementary information [file biolopen-7-034397-s1.pdf]

Table S1: Ingredient and chemical composition of experimental diets (A, B, D) in reared *Oncorhynchus mykiss*.

	A	B*	D
Ingredient composition (g kg^{-1})			
Marine protein / oil	220 / 80	560/120	400/100
Plant protein / oil	560 / 20	310 / -	340 / 60
Wheat	110		90
Vitamin & mineral premix	10	10	10
ratio: (marine protein / marine oil)	2.7	4.7	4.0
ratio: (plant protein / plant oil)	28.0	-	56.7
Chemical composition (%)			
Crude protein	45	44	45
Total lipid	22	23	21
Ash	7.1	10	8
GE (kJ.kg^{-1}DM)	21.5	21.5	21.7

* **Mente, E., Pierce, G. J., Antonopoulou, E., Stead, D. and Martin, S. A. M.** (2017). Postprandial hepatic protein expression in trout *Oncorhynchus mykiss* a proteomics examination. *Biochemistry and Biophysics Reports* **9**, 79-85.

Table S2. Core and most abundant (cumulative relative abundance per treatment >80%) operational taxonomic units (OTU) found in the gut *Oncorhynchus mykiss*. **Purple**: core and most abundant OTUs; **Red**: core OTUs; **Blue**: most abundant OTUs; ≈: tentative phylogenetic affiliation.

OTU	Closest relative	Similarity (%)	GenBank accession No	Habitat of origin	Reference	Closest genus (% similarity)
0001	Clone TP-2 (Tenericutes)	95.8	DQ340193	*Gillichthys mirabilis* (mudsucker, estuarine fish) gut	Bano et al. (2007)	*Mycoplasma* (92%)
0002	TTGE gel band N123 (≈Firmicutes)	100.0	JN185158	*Oncorhynchus mykiss* gut	Unpublished	*Acetanaerobacterium* (93%)
0019	Clone P-H6 (Bacillales)	100.0	HQ897587	Atlantic salmon (*Salmo salar* L.) intestine	Unpublished	*Bacillus* (99%)
0027	Clone A292_NCI	100.0	FJ456668	*Notothenia coriiceps* (Southern Ocean fish) intestinal content	Ward et al. (2009)	*Photobacterium* (100%)
0025	Clone OTU250	100.0	KC120615	Farmed adult turbot (*Scophthalmus maximus*) gastrointestinal tract	Xing et al. (2013)	*Vulcaniibacterium* (100%)
0031	Clone WTB_O14	100.0	EU009832	Turkey cecum	Scupham et al. (2008)	*Lactobacillus* (100%)
0033	Clone N4-2	100.0	KM974899	*Camponotus japonicus* (Japanese carpenter ant) gut	Unpublished	*Moraxella* (100%)
0038	*Lactobacillus salivarius* (Lactobacillales)	100.0	KR492877	Chicken cloace	Unpublished	*Lactobacillus* (100%)
0039	Clone AcqueII_100 (Rhizobiales)	100.0	HF912305	Process white water of a paper mill	Unpublished	*Microviriga* (98%)

Biology Open • Supplementary information

0047	Cone P-H6 (Bacillales)	100.0	HQ897587	Atlantic salmon (*Salmo salar*) intestine	Unpublished	*Bacillus* (99%)
0058	*Sphingobium yanoikuyae* (Sphingomonadales)	100.0	KM871866	Puffer fish digestive gland	Unpublished	*Sphingobium* (100%)
0069	*Lactobacillus delbrueckii* subsp. *bulgaricus* (Lactobacillales)	100.0	KJ729046	*Labeo rohita* (rohu/roho labeo, river water fish) intestine	Unpublished	*Lactobacillus* (100%)
0078	Clone Woods-Hole_a1939	100.0	KF799397	*Ciona intestinalis* (sea squirt) gut	Dishaw et al. (2014)	*Photobacterium* (99%)
0087	*Weissella* sp. (Lactobacillales)	100.0	KM088091	*Tenebrio molitor* (yellow mealworm beetle) larvae guts	Unpublished	*Weissella* (100%)
0100	Clone Comp5-2	100.0	KF911273	Cow manure composting	Unpublished	*Pseudoxanthomona* (100%)
0003	*Pseudomonas* sp. (Pseudomonadales)	100.0	KF366100	*Danaus plexippus* (overwintering butterflies) midgut	Unpublished	*Pseudomonas* (100%)
0004	Clone 25	100.0	DQ889971	Juvenile Atlantic salmon (*Salmo salar*) digestive tract	Navarrete et al. 2009	*Chroocidiopsis* (90%)
0005	*Cetobacterium somerae* 23 (Fusobacteriales)	97.9	HG326498	*Siganus canaliculatus* (rabbitfish, coral reef) gut	Unpublished	*Cetobacterium* (98%)
0006	Bio-material L100	99.6	HG966676	*Pisum sativum* subsp. *elatius* (wild pea) chloroplast	Unpublished	*Photobacterium* (100%)

Biology Open • Supplementary information

0007	*Photobacterium phosphoreum* (Vibrionales)	100.0	KU204883	Host: *Salmo salar* (Atlantic salmon)	Unpublished	*Photobacterium* (100%)
0008	*Weissella confuse* (Lactobacillales)	100.0	LC127180	Human faeces	Unpublished	*Weissella* (100%)
0009	Clone T-RFLP_clone_K44 (≈Tenericutes)	100.0	KP780113	Chicken caeca	Witzig et al. (2015)	*Chroocidiopsis* (91%)
0010	*Pseudomonas brenneri* (Pseudomonadales)	100.0	KU750791	Rhizosphere from *Lepidium meyenii* (maca)	Unpublished	*Pseudomonas* (100%)
0011	Clone Sch1000_2	99.6	HE586962	Freshwater fish gut	Unpublished	*Lactobacillus* (100%)
0012	Clone FecI096 (Lactobacillales)	100.0	KM244870	Faecal matter of pigs under indoor system	Unpublished	*Lactobacillus* (100%)
0013	*Photobacterium phosphoreum* (Vibrionales)	100.0	KJ817442	Atlantic salmon (*Salmo salar*) faeces	Hatje et al. (2014)	*Photobacterium* (100%)
0014	*Pseudomonas nitroreducens* (Pseudomonadales)	100.0	JX987715	Shrimp and crab farming pond water	Unpublished	*Pseudomonas* (100%)
0015	Clone OTU0162 (Pseudomonadales)	100.0	KM059059	*Bactrocera minax* (Chinese citrus fly) gut and reproductive organ	Wang et al. (2014)	*Pseudomonas* (100%)
0016	*Plesiomonas shigelloides* (Enterobacteriales)	100.0	DQ822763	Intestinal bacteria of freshwater salmon *Salmo salar* and sea trout *Salmo trutta trutta* and diet	Skrodenytė-Arbačiauskienė et al. (2008)	*Plesiomonas* (100%)

Biology Open • Supplementary information

0017	*Acinetobacter* sp. (Pseudomonadales)	100.0	KU159259	Wetland	Unpublished	*Acinetobacter* (100%)
0018	*Pseudomonas* sp. (Pseudomonadales)	100.0	KF366100	Midgut of *Danaus plexippus* (overwintering butterflies)	Unpublished	*Pseudomonas* (100%)
0021	*Streptococcus equinus* (Lactobacillales)	100.0	LC145574	Cow faces	Unpublished	*Streptococcus* (100%)
0023	*Herbaspirillum huttiense* subsp. *putei* (Burkholderiales)	100.0	NR_114068	Well water	de Souza et al. (2013)	*Herbaspirillum* (100%)
0024	*Bacillus* sp. (Bacillales)	99.6	KT751303	Soil	Unpublished	*Bacillus* (100%)
0030	*Escherichia coli* (Enterobacteriales)	100.0	KT898132	Host: swine	Unpublished	*Escherichia* (100%)
0056	*Pedobacter* sp. (Sphingobacteriales)	100.0	KM187459	*Pseudacris crucifer* (spring peeper, frog) skin	Walke et al. (2015)	*Pedobacter* (100%)
0063	*Chryseobacterium pallidum* (Flavobacteriales)	99.6	KU362282	Soil	Unpublished	*Chryseobacterium* (100%)

Biology Open • Supplementary information

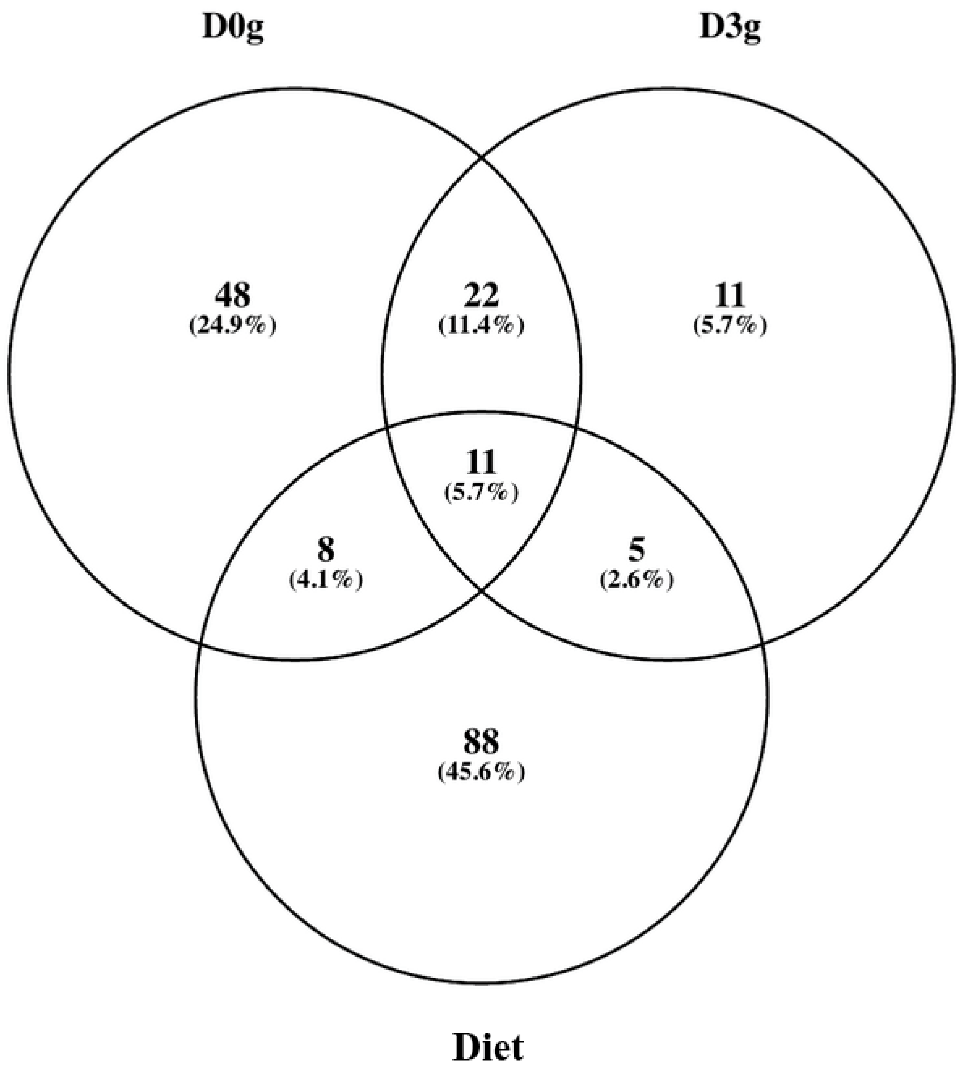

Figure S1. Shared operational taxonomic units (OTUs) between the diet D and the midgut (g) of reared *Oncorhynchus mykiss* before feeding at 0 h (D0g) and at 3 h (D3g) after feeding diet D.

Biology Open • Supplementary information
